# Human Health Risk Assessment from Mercury-Contaminated Soil and Water in Abu Hamad Mining Market, Sudan

**DOI:** 10.3390/toxics12020112

**Published:** 2024-01-29

**Authors:** Ahmed Elwaleed, HuiHo Jeong, Ali H. Abdelbagi, Nguyen Thi Quynh, Tetsuro Agusa, Yasuhiro Ishibashi, Koji Arizono

**Affiliations:** 1 Graduate School of Environmental and Symbiotic Sciences, Prefectural University of Kumamoto, Tsukide 3-1-100, Higashi-ku, Kumamoto 862-8502, Japan; g2175005@pu-kumamoto.ac.jp (A.E.);; 2 Mining Engineering Department, Faculty of Engineering, University of Khartoum, Gamma Ave., Khartoum P.O. Box 321, Sudan; ali.abdalbagi@uofk.edu; 3 Faculty of Environmental and Symbiotic Sciences, Prefectural University of Kumamoto, Tsukide 3-1-100, Higashi-ku, Kumamoto 862-8502, Japan; jeong-hui@pu-kumamoto.ac.jp (H.J.); yisibasi@pu-kumamoto.ac.jp (Y.I.); 4 School of Pharmaceutical Sciences, Kumamoto University, 5-1 Oe-honmachi, Chuo-ku, Kumamoto 862-0973, Japan

**Keywords:** mercury, ASGM, hazard quotient, health risk assessment, Sudan

## Abstract

Artisanal and small-scale gold mining (ASGM) poses a significant global threat due to mercury emissions and resulting health hazards. This study focuses on assessing these risks in the Abu Hamad ASGM community in Sudan. Utilizing the Mercury Analyzer 3000 (NIC), analyses of twelve soil samples (including one tailings sample) and seven water samples revealed the highest concentrations near amalgam burning locations: 34.8 mg/kg in soil (S06) and 3.26 µg/L in water (W03). Concentrations decrease with distance, with soil near burning exceeding tailings (S05 = 19.0 mg/kg). Hazard quotients indicate mercury vapor inhalation as the primary exposure route from soil, with the Hazard Index reaching 5.34 for adults and 33.4 for children close to amalgam burning sites. Water samples generally pose little risk except for W03, where children face potential danger via ingestion (HI = 1.74). These findings emphasize the urgent need for adopting retorts and eco-friendly practices to reduce mercury emissions and protect ASGM communities.

## 1. Introduction

Mercury has been extensively studied in environmental research among heavy metals, mainly due to its toxicity and high mobility [1]. This classification has led to its recognition as the third most hazardous pollutant by the United States Agency for Toxic Substances and Disease Registry (ATSDR) [2,3,4]. Despite its toxicity, artisanal miners prefer using mercury for gold extraction through amalgamation, given its simplicity and lower cost [5,6].

Gold, being the most extensively mined element, has seen a surge in global mining activity due to its increasing price, attracting over 20 million miners worldwide [7,8]. It also constitutes 50% of the estimated 40 million miners globally engaged in mining various minerals [9,10]. This substantial increase in miners and gold production contributes to approximately 37% of total mercury emissions, making it the largest anthropogenic source [11]. In Sudan alone, the number of artisanal miners is estimated to exceed one million [12], with ASGM communities comprising around 2 million individuals, as indicated by the Minamata Initial Assessment in Sudan [13].

Gold recovery by amalgamation is an inefficient process, in which only about 30% of gold is recovered [14]. Therefore, a substantial amount of mercury is lost to the environment. In the course of these procedures, mercury can inadvertently evaporate into the atmosphere and subsequently settle in nearby soil and aquatic ecosystems [15]. Mercury can also be transported to aquatic systems through leaching and soil erosion mechanisms [16].

Individuals residing or working in the vicinity of ASGM communities may face exposure to mercury vapor [17]. Consumption of food cultivated in mercury-polluted soils can pose significant health effects [18,19,20].

Mercury is an extremely hazardous element, and there is no recognized safe threshold for exposure [21]. Even at low concentrations, it is toxic to humans, affecting the central human nervous system and causing other detrimental effects as well [22]. Its toxicological impacts span cellular, cardiovascular, hematological, pulmonary, renal, immunological, neurological, endocrine, reproductive, and embryonic systems [23].

Sudan is among the largest mercury emitters in Africa, particularly from ASGM activities, driven by growing of miners. Among many potential hotspots of mercury in Sudan, Abu Hamad, a region in the River Nile State, was chosen for this study, since it is recognized for its historical significance in gold mining as one of the earliest and largest ASGM communities in the country. A few research studies have explored mercury exposure in ASGM and its associated health risks in Abu Hamad. One of the few studies highlighted elevated serum mercury levels among traditional gold miners from August 2012 to November 2014 [24]. Another study focused on urinary mercury concentrations in individuals occasionally exposed to mercury during artisanal gold mining, revealing alarming levels in 98% of participants [25].

The evaluation of mercury in soil, as an important pool for mercury accumulation [26], as well as water, was not comprehensively evaluated in many mercury hotspots in Sudan. Therefore, this study aims to: (1) study the spatial distribution of mercury in soil and water samples, and (2) the potential environmental and human health risk in the Abu Hamad ASGM area. This first-ever investigation in Abu Hamad ASGM community will provide valuable insights into the extent, distribution and potential health impacts of mercury contamination within the ASGM community.

## 2. Materials and Methods

### 2.1. Study Area

Abu Hamad is a city located in northern Sudan, in the River Nile State. It has been a significant center for gold mining, predating the nationwide gold rush that began in Sudan in 2009. Due to its proximity to the River Nile and its fertile agricultural land, it has attracted substantial agricultural investments. Since 2009, with the discovery of gold deposits, many miners have migrated to the city, and it has since become a thriving center for artisanal mining. Subsequently, numerous mining companies have initiated operations in the region. Furthermore, previously discarded tailings have become the lifeblood of a novel economic ecosystem, built upon the ingenuity of extracting residual gold. These gold-rich tailings have become attractive for the cyanide recovery businesses, which purchase them from ASGM processing centers and employ various cyanide techniques, including Carbon-In-Leach (CIL), Carbon-In-Pulp (CIP), heap leaching, and VAT leaching, for gold recovery.

To manage the spread of tailings within the residential area of Abu Hamad, the government of Sudan intervened and relocated all ASGM processing operations to controlled areas monitored by state officials and police [13]. These areas are known as Mining Markets and provide essential services for miners, including ore milling, goldsmith shops where they can sell their produced gold particles, healthcare centers, grocery stores, restaurants, money transfer services, and fuel stations, among others. The Abu Hamad Mining Market is one of the 73 mining markets across Sudan and holds significant importance [13]. It caters to numerous ASGM production sites in the vicinity. The Abu Hamad Mining Market comprises two gates. The southern gate, leading to the city of Abu Hamad, allows people’s movement and the entry of trucks carrying supplies such as fuel and water. The trucks laden with ore or amalgamation tailings pass through the northern gate, destined for the cyanide recovery facilities located north of the ASGM market.

In this study, one of the hypotheses under investigation pertains to the unpaved conditions of the road, which may result in trucks carrying tailings inadvertently spilling them route to the cyanide facilities situated north of the ASGM area. To explore the possibility of such tailing spills leading to land contamination by mercury, sampling was also conducted in the northern region of the ASGM.

Considering the arid climate and scarce surface water in the study area, wind-driven mercury dispersal emerged as a key concern. The research delved into the potential impact of wind, which could disperse mercury in the area, directly contaminating agricultural lands or nearby irrigation canals, such as the Kihiala irrigation canal situated to the south of the ASGM area.

### 2.2. Sampling of Soil and Water Samples

**Figure 1:**
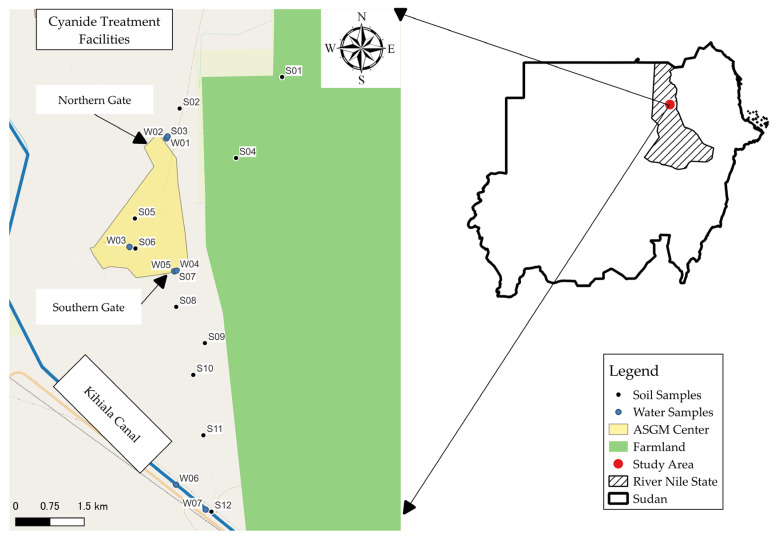
Sampling locations in the study area.

Sampling was conducted in January 2023. Twelve soil samples were collected from various locations within the study area, as shown in Figure 1. These soil samples represented different sampling points, including one sample of tailings (S05).

Soil samples were collected from a depth of 0–25 cm using manual shovels and scoops. The sampling points meticulously selected to cover the entire spatial extent of the study area, particularly in parallel to the two gates of the Abu Hamad Mining Market to cover the extent of mercury pollution.

Regarding water samples, as the area experiences an arid climate with limited rainfall, there were no surface water sources near the ASGM area. Instead, all the water used in the ASGM market is transported from the River Nile, located approximately 10 km away from the ASGM community, via water tanker trucks.

Out of the 7 water samples collected for analysis, two samples were obtained from the Kihiala irrigation canal (W06 & W07), which originates from the River Nile and serves as an irrigation source, as shown in Figure 1. The remaining 5 samples were collected from different water storage conditions and served various end-use purposes within the ASGM area. These samples represented variations in storage conditions and usage within the ASGM community.

### 2.3. Analytical Methodology

#### 2.3.1. Pretreatment of Samples

All water samples underwent on-site acidification with HNO_3_ to maintain a pH level below 2. The acidified samples were meticulously sealed and packed in plastic bottles covered by a thin film and placed in a cooler box for secure transportation to the laboratories. Upon arrival, the samples were preserved at temperatures below +4 °C until the time of analysis. For soil samples, an on-site sieving process was employed to eliminate debris and non-soil particles. The sieved samples were then air-dried at room temperature and further processed by sieving with a 150 µm mesh to homogenize them.

#### 2.3.2. THg Determination

The determination of total mercury concentration in water samples adhered to the established protocol outlined in the United States Environmental Protection Agency (USEPA) method no. 245.1 [27], and analyzed using Mercury Analyzer (MA-3000) (Nippon Instruments Corporation, Tokyo, Japan). For soil samples, approximately 30 mg of air-dried samples were placed in sample boats in triplicates and measured using a mercury analyzer (MA-3000).

#### 2.3.3. Quality Assurance

Stringent quality control measures were implemented to ensure the reliability of mercury analysis in water samples. The Limit of Detection (LOD) was determined using the Method Detection Limit (MDL) method, resulting in an MDL value of 0.005697 µg/L. The subsequent Limit of Quantification (LOQ) was established as 0.05697 µg/L, calculated as 10 times the SD. Accurate calibration curves were established for both low and high concentrations. The low calibration curve equation (y = 210.77x − 0.0543) demonstrated excellent linearity (R^2^ = 0.9995) within the range of 0–5 ng. Similarly, the high calibration curve equation (y = 272.86x − 0.9365) exhibited high linearity (R^2^ = 0.9931) over the range of 0–30 ng. To validate the accuracy of the analytical method, a recovery test was conducted by adding a known amount of mercury to the sample, with a duplicate process performed. Recovery tests yielded an average of 88.5%, and the coefficient of variation was calculated at 2.3%. Individual water sample analyses was performed in triplicate, and all samples exhibited coefficients of variation below 5%.

For soil samples, the analysis was conducted in triplicate, resulting in a coefficient of variation of less than 9%. Certified Reference Materials (CRM) were used, with NMIJ CRM 7302-a from the National Metrology Institute of Japan, designed for trace elements in marine sediment. The obtained value (0.511 ± 0.011) mg/kg corresponded to a recovery rate of (98.45 ± 2.19%), validating the accuracy and precision of the analytical method employed in this study.

### 2.4. Risk Assessment

Risk assessment involves evaluating the likelihood and potential magnitude of adverse events on health, safety, or the environment within a specific timeframe [28]. The assessment of health risks associated with individual toxic metals typically considers two primary factors: the slope factor (SF) for carcinogenic risk and the reference dose (RfD) for non-carcinogenic risk [29]. In this study, the RfD is utilized for the risk characterization.

The USEPA Exposure assessment model [30] was applied to determine the Average Daily Intake (AvDi) of mercury in soil and water samples (mg/kg-body weight/day). This assessment considered exposure through various routes for soil, including ingestion, dermal contact, inhalation of particulates containing mercury [31], and inhalation of mercury vapors (volatile mercury) [31]. Additionally, two exposure routes were considered for water samples: ingestion and dermal contact, as represented in Equations (1)–(4). Detailed input parameters for this study can be found in Table 1.

(1) $$AvDi\left(ing-soil,ing-water\right)=\frac{Cs,w*IRs,w*ED*EF*CF}{BW*AT}$$

(2) $$AvDi\left(der-soil,der-water\right)=\frac{Cs,w*SA*AF*ABS*ED*EF*GI*CF}{BW*AT}$$

(3) $$AvDi\left(vap\_soil\right)=\frac{Cs,w*IRa*ED*EF}{VF*BW*AT}$$

(4) $$AvDi\left(inh\_soil\right)=\frac{Cs,w*IRa*ED*EF}{PEF*BW*AT}$$

(5) HQ=AvDi/RfD

(6) HI=Σ(HQ1+HQ2+HQ3+HQ4)

For non-carcinogenic effects characterization, the Hazard Quotient (HQ) was computed using Equation (5). An HQ exceeding 1 indicates an unacceptable risk of adverse non-carcinogenic effects on health, while an HQ below 1 signifies an acceptable level of risk. In cases involving multiple exposure routes, the Hazard Index (HI) is employed (Equation (6)), where an HI above 1 indicates an unacceptable risk, and an HI below 1 indicates an acceptable risk level [29,31].

### 2.5. Statistical Analysis

Statistical analyses involved the use of the Mann–Whitney U test to compare medians between upwind and downwind samples, while the Spearman correlation was applied to assess the relationship between water and soil samples from the same locations. These analyses were performed using IBM SPSS 26 (SPSS Inc., Chicago, IL, USA). Graphs were generated using OriginPro 2024 (10.1.0.170) and Microsoft Excel 2019, and the map was created using Quantum GIS (QGIS 3.32). Meteorological data were obtained from the National Aeronautics and Space Administration (NASA) Langley Research Center (LaRC) Prediction of Worldwide Energy Resource (POWER) Project, funded through the NASA Earth Science/Applied Science Program, and processed using Microsoft Excel.

## 3. Results

### 3.1. Mercury Content Analysis

**Figure 2:**
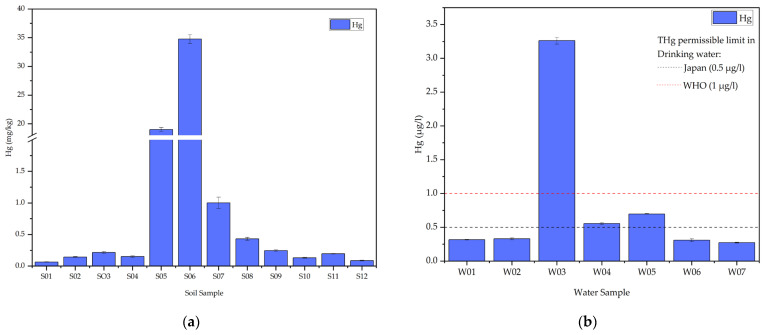
(**a**) Total mercury (THg) content in soil samples; (**b**) Total mercury (THg) content in water samples.

In the assessment of mercury distribution within the ASGM community, the analysis of soil samples revealed notable variations in mercury content, as depicted in Figure 2a. The tailings sample (S05) exhibited a lower mercury concentration compared to soil samples collected near goldsmith shops where amalgam is burned (S06). Particularly, the highest mercury content was identified in S06 (34.8 mg/kg), located in close proximity to goldsmith shops, followed by S05 (19.0 mg/kg), a tailings sample. Conversely, the lowest mercury concentration was observed in S01 (0.06 mg/kg), situated furthest north of the ASGM market. Since no Sudanese mercury background levels exist, the background level proposed by Reimann and De Caritat [34] (0.04 mg/kg) is employed for the comparison of soil samples, all of which exceed this specified value. It is important to note that many countries have established different background levels of mercury based on land use. In this study, the ASGM center is considered an industrial area; therefore, all samples are expected to exceed the background values for the Earth’s crust.

Simultaneously, water samples exhibited varying total mercury concentrations, with the highest recorded at W03 (3.26 µg/L), were located at the center of the ASGM community, near amalgam burning shops, where it exceeded the established safety thresholds as per the guidelines set forth by the World Health Organization (WHO) [35], Sudanese Guidelines and standards for drinking water [36], Drinking Water Quality in Japan [37], and the United States Environmental Protection Agency (USEPA) [38]. While W04 (0.56 µg/L) and W05 (0.7 µg/L) surpass the drinking water quality standards of specific countries like Japan, which sets a limit of 0.5 µg/L, they still fall below the standards set by WHO, Sudan, and USEPA, as shown in Figure 2. In contrast, the lowest mercury concentration in water was identified at W07 (0.27 µg/L), sourced from the Kihiala irrigation canal south of the ASGM community, as shown in Figure 2b.

A significant positive relationship between soil and water samples collected from the same locations was observed (*p* < 0.001), and this association was further confirmed using Spearman’s correlation (r = 1). The utilization of these statistical techniques accounted for the presence of outliers in the relationship.

### 3.2. Spatial Distribution of the Mercury in Soil and Water Samples

Further examination of spatial patterns revealed a decreasing trend in both soil and water samples as distance increased from the center of the ASGM area. Notably, the lowest concentrations were found in S12 (0.09 mg/kg) in the south direction and S01 (0.06 mg/kg) in the north direction.

Wind direction plays a significant role in mercury distribution [39]. Samples taken upwind (S01–S04) displayed a lower median mercury concentration (0.146) mg/kg compared to downwind samples (S07–S11) with a median concentration of (0.219) mg/kg. Due to non-normal distribution and unequal variances, the Mann–Whitney U Test was employed for comparing the median total mercury concentrations between groups (*p*-value 0.248 > 0.05). Consequently, while there is a trend of mercury accumulation in the downwind direction, statistical significance was not established.

### 3.3. Human Health Risk Assessment

Among the exposure routes examined, ingestion of water and soil particles alongside mercury vapor inhalation were identified as the primary culprits behind mercury-related health risks. Notably, the center of the ASGM market was the sole location where the Hazard Index (HI) exceeded 1.

Adults face significant health risks only in the center of the ASGM market, near tailing sites (S05) and amalgam burning areas (S06). Here, the Hazard Index (HI) in soil samples soars above 1 (2.92 and 5.34, respectively) due to the inhalation of mercury vapor (Table 2). These sites, located in the center of the ASGM market, included a tailing sample (S05) and a soil sample taken near the amalgam burning area (S06). All water samples for adults registered HI values below 1 in all areas (Table 3).

Children face similar elevated HI values in the ASGM market center (S05 and S06), even exceeding adults, reaching alarming heights of 18.59 and 33.98 (Table 4). This significant risk comes from both ingestion and vapor inhalation, with S05 showing an ingestion HQ of 0.81 and a vapor inhalation HQ of 17.6, while S06 has higher values at 1.48 and 32.2, respectively. Interestingly, S07, while just below an HI of 1 (0.98), still exposes children to concerning levels of mercury vapor.

Water samples maintained HI values below 1 for children, except for W03, where the HI reached 2.4 via ingestion. Ingestion dominated as the primary exposure route for children in water samples (Table 5).

## 4. Discussion

### 4.1. Mercury Level Distributions in the Study Areas

Mercury’s fate within the ASGM area proved more intricate than predicted, where a higher total mercury content in amalgamation tailings was a central expectation, given the direct contact between mercury and ore in the whole-ore-amalgamation (WOA) process employed in Sudan [13]. Unexpectedly, tailings (S05) contained less mercury (19.0 mg/kg) than soil near burning shops (S06) (34.8 mg/kg), as shown in Figure 2a.

This unexpected result suggests a complex influence of the amalgamation and burning processes on mercury distribution. Specifically, the elevated mercury concentration in the soil near the amalgam burning area (S06) may be attributed to factors such as inadequate use of retorts in most amalgam burning shops. The absence of retorts can contribute to the dispersion of mercury, potentially contaminating the surrounding soil and water samples. Significantly, this pattern is also observable in water sample W03, as shown in Figure 2.

The close linkage between soil and water contamination became especially apparent in locations like S06 and W03, where the soil sample boasted a substantial 34.8 mg/kg of mercury, mirrored by a 3.26 µg/L concentration in the corresponding water sample. This statistically significant correlation (Spearman r = 1, *p* < 0.001) held true across other pairings, like S07 and W05, S03 and W03, and S12 and W07, highlighting the interconnectedness of mercury pollution in the study area. Consistent with other studies, areas closest to burning exhibited the highest concentrations [40,41,42], suggesting an influence from mercury vapors resulting from the amalgam burning process. However, it is essential to acknowledge certain study limitations, including the lack of data on mercury concentrations in the suspended phase and the absence of temporal variations in water and soil sample concentrations. Addressing these gaps is crucial for a more comprehensive understanding of mercury fate across various environmental compartments in the study area.

**Figure 3:**
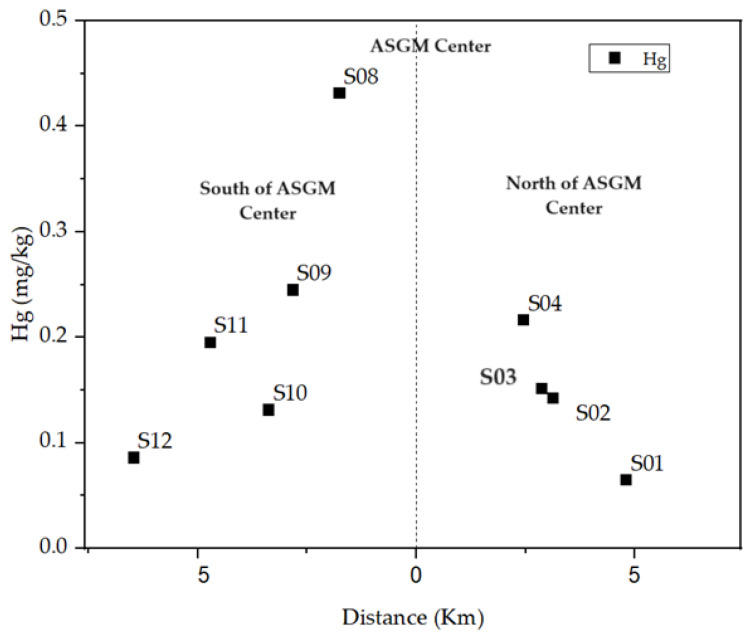
Decrease of THg concentration with distance.

The distribution of total mercury in the study area displayed a decreasing trend, aligning with findings from various studies [43,44,45]. This decline was observed in both north and south directions from the center of the amalgam burning site, as shown in Figure 3. Several hypotheses were considered to understand the mechanisms contributing to mercury dispersal in the area, including wind direction [46,47], surface water and rainfall [46], and the potential spillage of tailings from trucks transporting amalgamation tailings to cyanide recovery facilities located north of the ASGM community [48].

Surface water transport of mercury from uncovered amalgamation tailings was deemed unlikely due to the prevailing desert climate and limited rainfall in the study area (50 mm/year) [49]. Consequently, the primary considerations were wind direction and the transportation activities through the north and south gates of the ASGM community.

**Figure 4:**
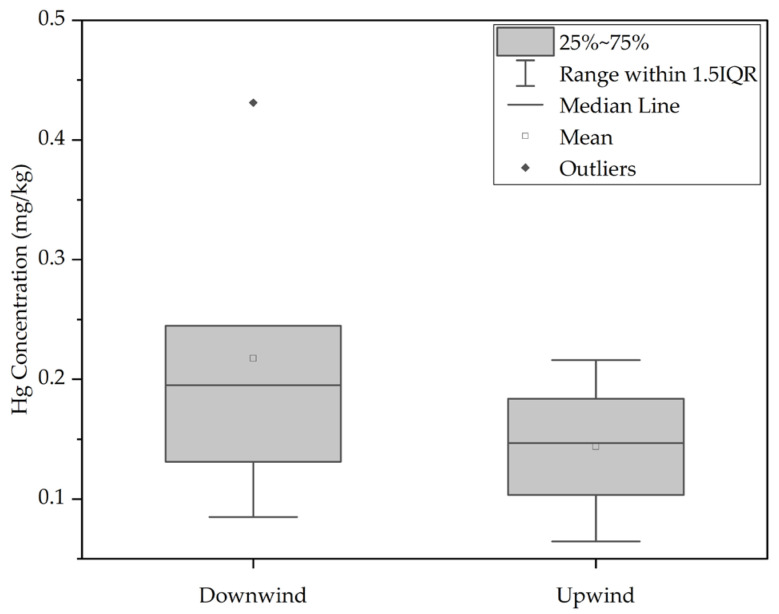
Comparison between Hg concentration in soil collected downwind and upwind.

Despite expectations that the north gate, used by trucks carrying tailings, would accumulate more mercury, results indicated a decrease in mercury content in both north and south directions, where the furthest north (S01) and south (S12) points exhibited the lowest mercury accumulation. The median mercury concentration in soil samples from the southern areas (0.219 mg/kg) was relatively higher than those from the north (0.146 mg/kg) as shown in Figure 4, but statistical analysis found no significant difference.

**Figure A1:**
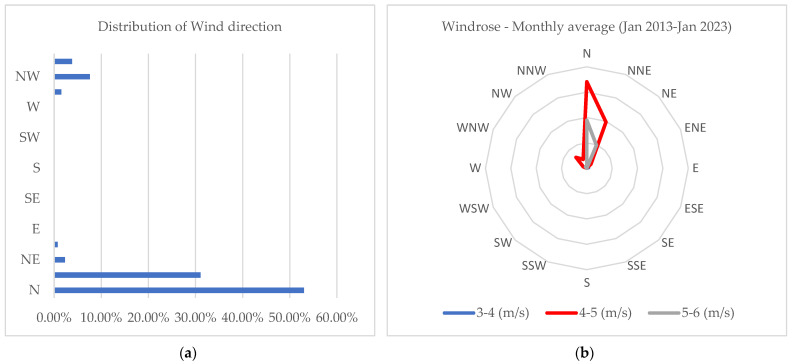
Distribution of wind direction and windspeed at the study area: (**a**) Distribution of wind direction (January 2013–January 2023); (**b**) Monthly Windrose (January 2013–January 2023).

The wind direction, prevailing from the north (Figure A1), was anticipated to influence the accumulation of mercury in the southern areas. However, the lack of statistical significance between the median values of the samples upwind and downwind suggested that other factors might contribute to mercury accumulation in the southern regions.

One potential factor that may explain the higher median in samples south of the ASGM community is the traffic density at the southern gate, which is busier with vehicles carrying fuel, water, and other materials. This elevated traffic may contribute to higher mercury concentrations in the southern areas, challenging the assumption that trucks in the north are the primary source of soil pollution. While trucks were initially suspected, wind and traffic density emerged as a tangled web influencing mercury accumulation in the ASGM soil.

### 4.2. Health Risk Assessment

Mercury toxicity varies with dosage; a substantial acute exposure to elemental mercury vapor can result in severe pneumonia, with extreme cases leading to the conclusions found in the work presented in [22,50]. Conversely, low levels of chronic exposure to elemental or other forms of mercury may manifest with more subtle symptoms and clinical findings [22,51].

In this study, the primary exposure route to elemental mercury for humans is through the inhalation of mercury vapor, a scenario well-documented in literature [52]. The high volatility of mercury allows it to evaporate easily, making inhalation a significant mode of exposure [53]. However, the inhalation of suspended particles is considered nearly negligible when compared to other exposure routes [53,54]. Subsequently, the risk is compounded by the ingestion of water and soil particles contaminated with mercury.

The ingestion of mercury-contaminated soils and water emerges as a noteworthy human health risk in the study area. It is important to note that while samples collected outside the ASGM area exhibit elevated mercury content, they do not pose a significant risk according to the human health risk assessment. In contrast, samples collected within the ASGM area, including both adults and children, indicate potential health risks, particularly at sampling points S05 and S06, where HI exceeds 1 at both sampling locations, representing about 16.67% of the total soil samples.

For adults, the only exposure route posing a danger is the inhalation of mercury vapor from the mercury-contaminated soils at sampling points S05 and S06, with HQvap values of 2.8 and 5.12, respectively. Similarly, these samples indicate potential hazard for children, with HQvap values of 17.6 and 32.2, respectively. Furthermore, the same samples (S05 and S06) recorded HQing values of 0.81 (S05) and 1.48 (S06), indicating that children face additional danger through ingestion. Notably, sample point S07 records an HI of 0.98, approaching 1, indicating that all soil samples within the ASGM area present potential human health risks for children. Children, due to their higher consumption per unit of body weight compared to adults, are particularly susceptible to the acute, subacute, and chronic effects of ingesting chemical pollutants [33].

Water samples showed no human health risks for adults, as they do not exceed HI > 1. In the case of children, only sample W03 poses a potential risk with an HI of 1.74 via the ingestion route. Ingestion is identified as the primary contributor to human health risksin water samples.

Despite some safe areas, it is crucial to implement mitigation measures to avert the consequences of mercury pollution. Children are more vulnerable to the effects of mercury exposure than adults due to their higher consumption per unit of body weight [33] and their developing neurocognitive systems [55]. Despite the fact that children are not typically present or allowed in the Abu Hamad Mining market, precautions are still necessary.

### 4.3. A Cross-Study Evaluation

Table 6 highlights a comparative analysis of various global studies, shedding light on diverse methodologies and outcomes. In a study in Brazil [56], the relatively lower mercury concentrations in aged tailings and exclusion of the assessment of vapor inhalation as exposure route in the study, led to significantly lower HI values. Conversely, in the study in Nigeria [57] and our study, the mercury concentrations in soil and tailings samples align in the same order of magnitude, yet variations in Health Risk Assessment parameters yield differing risk values. In a study in Ghana [58], similar parameters to our study were employed, except for a twofold higher ingestion rate for soil in informal residential areas for children. This adjustment results in children’s Health Index (HI) values twice those in our study. Furthermore, while the Ghanaian evaluation employed mean values, the actual range is an order of magnitude higher (0.2 to 410 mg/kg) than in our study, explaining the higher HI values for adults.

Regarding water samples, concentrations are comparable between our study and a study in Ecuador [59], but the maximum concentration in Ecuador is three times higher, contributing to elevated HI values. In Ghana [60], water sample concentrations are two orders of magnitude higher than in other studies, resulting in HI values at the same order of magnitude but significantly higher. It is crucial to note the inherent variability in Health Risk Assessment values across studies, arising from differences in evaluation parameters.

### 4.4. Reduction of Mercury Emissions

To effectively counter mercury’s impact, minimizing exposure routes, especially near burning sites, and implementing targeted interventions are crucial. The findings offer valuable insights for the development and implementation of sustainable interventions aimed at protecting the health of individuals within ASGM communities.

Despite the adoption of processing centers in many regions around the world, which has proven effective in limiting soil pollution within their vicinity [61], this study highlights that the population in ASGM communities continues to face mercury exposure through various routes. To alleviate mercury-related risks, several options are available, starting with the promotion of occupational health and safety awareness among miners, potentially facilitated through initiatives such as Street Theatre [62]. Additionally, promoting gravity concentration, a technique separating gold without mercury, can eliminate its harmful use and significantly reduce environmental contamination [63], or chemical processes like cyanidation [14]. Additionally, a combination of both [64], can contribute to reducing mercury levels.

The study underscores the significance of employing retorts and implementing environmentally friendly practices to decrease mercury emissions. This approach is crucial for mitigating the health risks associated with elevated concentrations found in soil and water samples.

## 5. Conclusions

Our investigation in Abu Hamad’s ASGM community unveils disturbing patterns of mercury distribution and alarming health risks, demanding immediate action to protect population within the community. Notably, soil proximal to amalgam burning sites exhibited concentrations surpassing initial expectations, indicative of intricate factors influencing mercury dispersion, notably the suboptimal use of retorts in these burning establishments. A robust correlation between soil and water samples underscored the impact of mercury vapors emanating from amalgam burning. Despite study limitations, such as the absence of suspended phase sampling, our findings markedly enhance comprehension of mercury exposure in ASGM communities.

The inhalation of mercury vapors and subsequent soil particle ingestion pose potential health risks for both adults and children. All samples within the ASGM community indicate a human health risk, characterized by Hazard Index (HI) values exceeding 1. Water samples, excluding those in close proximity to amalgam burning sites, do not pose a human health risk for both children and adults. However, water samples near amalgam burning sites register an HI exceeding 1 for children.

Future research will address data gaps by analyzing suspended mercury and temporal variations in soil and water concentrations. A nuanced exploration of multifaceted factors influencing mercury accumulation, encompassing wind direction and traffic density, will enrich our comprehension. The Health Risk Assessment underscores potential risks, accentuating the necessity for tailored interventions to safeguard the well-being of ASGM community members. Mitigation strategies, such as promoting retorts and gravity concentration techniques, should be prioritized to reduce mercury emissions and mitigate associated health risks.

## Figures and Tables

**Table 1:** Input parameters for evaluating Average Daily Intake (AvDi) and Hazard Quotient (HQ).

Parameters		Unit	Children	Adults	Reference
Cs	Total mercury contents in soil samples	mg/kg	-	-	This Study
Cw	Total mercury concentration in the water samples	mg/L	-	-	This Study
BW	Body weight	Kg	15	70	[32]
AT	Averaging time	Days	2190	10,950	[32]
EF	Exposure frequency	days/year	350	250	[32]
ED	Exposure duration	Years	6	30	[32]
IRs	Ingestion rate soil	mg/day	200	100	[32]
IRa	Ingestion rate air	m^3^/day	10	10.4	[32]
IRw	Ingestion rate water	L/day	2	3.45	[33]
GI	Gastrointestinal adsorption Rate	m^3^/kg	1	1	[32]
PEF	Particulate emission factor	m^3^/kg	1.30 × 10^9^	3.22 × 10^8^	[32]
VF	Volitilisation factor	m^3^/kg	8028.297	8028.297	[32]
SA	Surface area exposed skin	cm^2^	2100	13,110	[32]
AF	Skin aderence factor	mg/cm^2^/day	0.2	0.07	[32]
ABS	Dermal absorption factor	-	0.1	0.1	[32]
CF	Conversion factor	-	0.000001	0.000001	[32]
RfD_o_	Reference Dosage (Oral)	mg/kgbw/day	0.003	0.003	[32]
RfD_i_	Reference Dosage (Inhalation)	mg/kgbw/day	0.000086	0.000086	[32]
RfD_d_	Reference Dosage (Dermal)	mg/kgbw/day	0.003	0.003	[32]

**Table 2:** HQ and _e_AvDI values for soil (Adults).

Location	Sample	Hg	_e_AvDI (Ing)	HQ (ing)	_e_AvDI (der)	HQ (der)	_e_AvDI (inh)	HQ (inh)	_e_AvDI (vap)	HQ (vap)	HI
		mg/kg	(10^−6^)	(10^−3^)	(10^−7^)	(10^−3^)	(10^−11^)	(10^−7^)	(10^−6^)		
North of ASGM Area	S01	0.06	0.06	0.211	0.58	0.193	2.04	2.38	0.82	0.01	0.01
	S02	0.14	0.14	0.464	1.277	0.426	4.49	5.22	1.8	0.02	0.022
	S03	0.15	0.15	0.494	1.36	0.453	4.79	5.566	1.92	0.02	0.023
	SO4	0.22	0.21	0.705	1.942	0.647	6.83	7.95	2.74	0.03	0.033
ASGM Area	S05	19.0	18.61	62.02	170.746	56.915	600.94	698.76	241.02	2.8	2.922
	S06	34.8	34.02	113.385	312.162	104.054	1098.64	1277.49	440.64	5.12	5.341
	S07	1.00	0.98	3.268	8.996	2.999	31.66	36.82	12.7	0.15	0.154
South of ASGM Area	S08	0.43	0.42	1.406	3.871	1.29	13.63	15.84	5.46	0.06	0.066
	S09	0.25	0.24	0.798	2.198	0.733	7.73	8.99	3.1	0.04	0.038
	S10	0.13	0.13	0.428	1.178	0.393	4.15	4.82	1.66	0.02	0.02
	S11	0.2	0.19	0.636	1.751	0.584	6.16	7.17	2.47	0.03	0.03
	S12	0.09	0.08	0.28	0.772	0.257	2.72	3.16	1.09	0.01	0.013
Min		0.06	0.632	0.211	0.58	0.193	2.043	2.375	0.819	0.01	0.01
Max		34.8	340.156	113.385	312.162	104.054	1098.642	1277.491	440.645	5.124	5.341
Median		0.2055	2.012	0.6705	1.8465	0.6155	6.498	7.556	2.6065	0.0305	0.0315
SD		10.896	106.618	35.539	97.843	32.614	344.355	400.413	138.115	1.606	1.674

**Table 3:** HQ and _e_AvDI values for water (Adults).

Location	Sample	Hg mg/L	_e_AvDI (Ing)	HQ (ing)	_e_AvDI (der)	HQ (der)	HI
		(10^−3^)	(10^−6^)		(10^−10^)	(10^−6^)	
North of ASGM Area	W01	0.32	10.72	0.036	2.85	9.51	0.04
	W02	0.33	11.11	0.037	2.95	9.85	0.04
ASGM Area	W03	3.26	110.12	0.367	29.29	97.64	0.37
	W04	0.56	18.75	0.062	4.99	16.62	0.06
	W05	0.7	23.56	0.079	6.27	20.89	0.08
South of ASGM Area	W06	0.31	10.47	0.035	2.79	9.29	0.03
	W07	0.27	9.23	0.031	2.45	8.18	0.03
Min		0.27	9.23	0.031	2.45	8.18	0.03
Max		3.26	110.12	0.367	29.29	97.64	0.37
Median		0.329	11.11	0.037	2.95	9.85	0.04
SD		1.088	36.724	0.122	9.768	32.562	0.124

**Table 4:** HQ and _e_AvDI values for soil (Children).

Location	Sample	Hg	_e_AvDI (Ing)	HQ (ing)	_e_AvDI (der)	HQ (der)	_e_AvDI (inh)	HQ (inh)	_e_AvDI (vap)	HQ (vap)	HI
		mg/kg	(10^−6^)		(10^−7^)		(10^−11^)	(10^−7^)	(10^−5^)		
North of ASGM Area	S01	0.06	0.83	0.003	1.74	0.0006	3.18	3.7	0.51	0.06	0.06
	S02	0.14	1.82	0.006	3.82	0.0013	6.99	8.13	1.13	0.13	0.14
	SO3	0.15	1.94	0.006	4.07	0.0014	7.45	8.66	1.21	0.14	0.15
	S04	0.22	2.76	0.009	5.81	0.0019	10.63	12.36	1.72	0.2	0.21
ASGM Area	S05	19.0	243.12	0.81	510.55	0.1702	935.07	1087.29	151.41	17.61	18.59
	S06	34.8	444.47	1.482	933.39	0.3111	1709.5	1987.8	276.82	32.19	33.98
	S07	1.00	12.81	0.043	26.9	0.009	49.27	57.28	7.98	0.93	0.98
South of ASGM Area	S08	0.43	5.51	0.018	11.58	0.0039	21.2	24.65	3.43	0.4	0.42
	S09	0.25	3.13	0.01	6.57	0.0022	12.03	13.99	1.95	0.23	0.24
	S10	0.13	1.68	0.006	3.52	0.0012	6.45	7.5	1.04	0.12	0.13
	S11	0.2	2.49	0.008	5.24	0.0017	9.59	11.15	1.55	0.18	0.19
	S12	0.09	1.1	0.004	2.31	0.0008	4.23	4.91	0.68	0.08	0.08
Min		0.06	0.826	0.003	1.736	0.001	3.179	3.696	0.515	0.06	0.063
Max		34.8	444.471	1.482	933.389	0.311	1709.50	1987.796	276.815	32.188	33.981
Median		0.2055	2.629	0.0085	5.521	0.002	10.111	11.757	1.6375	0.1905	0.201
SD		10.896	139.314	0.464	292.559	0.097	535.822	623.049	86.764	10.089	10.651

**Table 5:** HQ and _e_AvDI values for water (children).

Location	Sample	THg (mg/L)	_e_AvDI (ing)	HQ (ing)	_e_AvDI (der)	HQ (der)	HI
		(10^−3^)	(10^−6^)		(10^−10^)	(10^−6^)	
North of ASGM Area	W01	0.32	5.08	0.169	8.53	2.84	0.17
	W02	0.33	5.26	0.175	8.83	2.94	0.18
ASGM Area	W03	3.26	52.13	1.738	87.58	29.19	1.74
	W04	0.56	8.87	0.296	14.91	4.97	0.3
	W05	0.7	11.16	0.372	18.74	6.25	0.37
South of ASGM Area	W06	0.31	4.96	0.165	8.33	2.78	0.17
	W07	0.27	4.37	0.146	7.34	2.45	0.15
Min		0.27	4.37	0.146	7.34	2.45	0.15
Max		3.26	52.13	1.738	87.58	29.19	1.74
Median		0.329	5.26	0.175	8.83	2.94	0.18
SD		1.088	17.384	0.580	29.207	9.734	0.579

**Table 6:** Comparative analysis of mercury concentrations and Health Risk Assessment parameters in various ASGM studies.

Country	Mercury Concentration		HI		Reference
			Children	Adults	
	Soil				
Brazil	Min (mg/kg)	0.05	5.18 × 10^−2^	5.6 × 10^−3^	[56]
	Max (mg/kg)	2.29	1.04	1.12 × 10^−1^	
Nigeria	Min (mg/kg)	0.002	0.044211	0.031	[57]
	Max (mg/kg)	20.99	4.863223	2.322	
Ghana	Min (mg/kg)	0.2	0.5	0.1	[58]
	Max (mg/kg)	43.3	76	10.9	
Sudan	Min (mg/kg)	0.06	0.06	0.01	This Study
	Max (mg/kg)	34.8	33.98	5.34	
	Water				
Ecuador	Min (µg/L)	0.6	0.29	0.1	[59]
	Max (µg/L)	9.9	2.56	0.9	
Ghana	Min (µg/L)	132	N/A	0.51	[60]
	Max (µg/L)	866	N/A	4.6	
Sudan	Min (µg/L)	0.27	0.15	0.03	This Study
	Max (µg/L)	3.26	1.74	0.37	

N/A for children [60]: Health risk assessment not conducted.

## Data Availability

Data are contained within the article.
